# Analysis of Iron and Iron-Interacting Protein Dynamics During T-Cell Activation

**DOI:** 10.3389/fimmu.2021.714613

**Published:** 2021-08-12

**Authors:** Megan R. Teh, Joe N. Frost, Andrew E. Armitage, Hal Drakesmith

**Affiliations:** ^1^ MRC Human Immunology Unit, MRC Weatherall Institute of Molecular Medicine, University of Oxford, John Radcliffe Hospital, Oxford, United Kingdom; ^2^ Haematology Theme, Oxford Biomedical Research Centre, Oxford, United Kingdom

**Keywords:** T-cell, iron, immunometabolism, adaptive immunity, iron deficiency, demethylation, Th17 cells, iron overload

## Abstract

Recent findings have shown that iron is a powerful regulator of immune responses, which is of broad importance because iron deficiency is highly prevalent worldwide. However, the underlying reasons of why iron is needed by lymphocytes remain unclear. Using a combination of mathematical modelling, bioinformatic analysis and experimental work, we studied how iron influences T-cells. We identified iron-interacting proteins in CD4+ and CD8+ T-cell proteomes that were differentially expressed during activation, suggesting that pathways enriched with such proteins, including histone demethylation, may be impaired by iron deficiency. Consistent with this, iron-starved Th17 cells showed elevated expression of the repressive histone mark H3K27me3 and displayed reduced RORγt and IL-17a, highlighting a previously unappreciated role for iron in T-cell differentiation. Quantitatively, we estimated T-cell iron content and calculated that T-cell iron demand rapidly and substantially increases after activation. We modelled that these increased requirements will not be met during clinically defined iron deficiency, indicating that normalizing serum iron may benefit adaptive immunity. Conversely, modelling predicted that excess serum iron would not enhance CD8+ T-cell responses, which we confirmed by immunising inducible hepcidin knock-out mice that have very high serum iron concentrations. Therefore, iron deficiency impairs multiple aspects of T-cell responses, while iron overload likely has milder effects.

## Introduction

Effective adaptive immunity is critical for the clearance of many pathogens. Activation of adaptive immune responses is metabolically demanding and reliant on nutritional factors such as glucose, amino acids and lipids (1). Growing evidence also implicates iron as a critical nutrient for adaptive immune responses (2). For instance, activating T-cells adopt an iron accumulatory phenotype characterised by upregulation of the iron acquisition proteins transferrin receptor (TFRC, CD71), solute carrier family-11 member-2 (SlC11A2) and SLC39A14, and suppression of the sole known iron exporter, Slc40a1 (3, 4). Further, children bearing an amino acid change in the transferrin receptor, that impairs cellular iron uptake, display severe combined immunodeficiencies featuring hypogammaglobulinemia and defective lymphocyte proliferation (5). Induction of hypoferremia (low serum iron) in mouse models impairs B and T-cell responses to both vaccination and influenza infection, while supply of iron to iron-deficient piglets improves vaccine responses (3). Effects of iron deficiency inhibiting, and iron supplementation enhancing vaccine responses in humans have also been reported (6, 7). Mechanistically, *in vitro* T-cell studies with iron starvation mediated *via* iron depleted media or the use of iron chelators demonstrate the importance of iron for cellular proliferation, activation and the production of effector molecules such as granzyme B (GZMB), granulocyte macrophage colony stimulating factor (GM-CSF) and interferon γ (IFN-γ) (3, 5, 8–10). While a profound role for iron in T-cell activation and function has been established, the specific dynamics of iron utilisation, and the key biological processes affected within T-cells remain unclear. Understanding how iron is used by T-cells may provide insight as to how iron modulation could be used to improve or attenuate immune responses in diverse contexts such as vaccination, infection or cancer.

Iron’s ability to act as an electron donor and acceptor is a characteristic often co-opted by cellular proteins for catalysis of reduction-oxidation (redox) reactions and oxygen binding (11). The central role of iron in cellular biochemistry was highlighted in a study by Andreini et al, which predicts that ~2% of human protein coding genes and ~6.5% of enzymes interact with iron (12). Protein interactions with iron can occur either directly with iron ions or *via* heme or iron-sulfur (Fe-S) cluster prosthetic groups (12). Iron ion binding proteins bind iron directly and are predominantly catalytic (12); many such proteins are 2-oxoglutarate (2-OG) dependent dioxygenases which mediate hydroxylation reactions involved in processes such as histone and DNA demethylation and collagen synthesis (12, 13). Meanwhile, heme cofactors consist of iron within larger porphyrin ring structures (14). Heme proteins, including hemoglobin are well known for oxygen binding, but are also involved in mitochondrial electron transfer and oxidative reactions involved in pathways such as prostaglandin synthesis, nitric oxide production and tryptophan metabolism (14). Finally, Fe-S clusters coordinate iron and sulfur atoms as [2Fe-2S], [3Fe-4S] or [4Fe-4S] structures in mammals (15). Fe-S cluster interacting proteins are extremely diverse and include proteins involved in Fe-S cluster synthesis itself, mitochondrial respiration and DNA synthesis (12, 15).

Using a previously published T-cell proteomics dataset from Howden et al. (4) that assesses T-cell protein content at 0h, 24h and 6 days post activation, and a list of iron interacting proteins derived from Andreini et al. (12), we aimed to understand how iron interacting proteins are differentially expressed during T-cell activation and differentiation. We identified processes enriched for iron interacting proteins in T-cells including demethylation, oxidative phosphorylation (OXPHOS), DNA synthesis and Fe-S cluster biogenesis and predict that T-cell iron requirements increase substantially post-activation. Using computational and experimental models we suggest that while iron deficiency may impair T-cell iron uptake, excess iron is unlikely to provide significant benefit to activating T-cells. Our analysis provides a unique approach to “immunometallomics” and provides insight as to the importance of iron in T-cell function.

## Methods

### Deriving a List of Iron Interacting Mouse Protein Homologues

Using the Uniprot IDs of human iron interacting proteins provided by Andreini *et al*, corresponding standard human gene names were identified using the Uniprot mapping tool (https://www.uniprot.org/uploadlists/) (16). To curate an equivalent list of mouse iron interacting protein homologues, the list of human iron interacting genes was input into the Ensembl BioMart tool (http://useast.ensembl.org/biomart/martview/893cea99357a57529ab65ce92c12e306) selecting for comparison to the Ensembl Genes 100 database, Human genes (GRCh38.p13) dataset (**Supplementary File 1**) (17). Filtering was completed by gene name using an external reference ID list and selecting for the attributes: gene stable ID, gene name, mouse gene stable ID, mouse gene name and gene description. This method was able to identify mouse homologues for the majority of human iron interacting proteins (349/398, 88%). In cases where gene matches were not identified by Ensembl, manual verification was completed and several more matches were identified (8 proteins). Some human iron interacting proteins were found to have no mouse homologues (23 proteins, ex//KDM4E, SCD5, NOX5, etc.) or poor gene annotation limited the identification of matches (18 proteins, ex//CYP2C, FADS2P1, DKFZp686G0638). To obtain further cofactor information regarding protein to iron atom stoichiometry, the Uniprot database was manually searched using the cofactor terms: 4Fe-4S, 3Fe-3S, 2Fe-2S, heme, Fe2+, Fe3+. Retrieved cofactor information was manually added to the list of iron interacting information

Notably, the iron interacting protein list does not include proteins that indirectly interact with iron such as TFRC which interacts with iron *via* intermediate contact with Tf. The resulting list of mouse iron interacting proteins is relatively comprehensive but likely does not include the complete set of iron interacting proteins. For instance, mouse iron interacting proteins with no corresponding human homologue or homologues that are poorly annotated would not be identified using this method. Alternatively, the possibility also exists that some mouse homologues of human iron interacting proteins may not themselves interact with iron.

### Identifying Iron Interacting Proteins in the Howden Dataset

Using bioinformatic methods, the list of mouse iron interacting proteins was compared against the complete list of proteins detected in the Howden dataset as well as individual lists of differentially regulated proteins as copy-number or concentration by T-cell sub-type (CD4-24h, CD8-24h, Th1, CTL). Matches were extracted and can be found in **Supplementary File 2**. In the case of the gene CIAO3, it was noted that the Howden dataset uses the alternative name NARFL. To ensure that NARFL was picked up by our analysis, NARFL was added as an alternative name for CIAO3 in our list of iron interacting proteins.

### Pathway Enrichment Analysis

Pathway enrichment for the genes of interest were analysed using unranked lists using the gProfiler algorithm, selecting for the gene ontology (GO) biological processes (18). Term size was limited to 3-500 genes and the significance threshold used was a Benjamini-Hochberg false discovery rate set at <0.05. Only gene intersections greater than 4 were plotted.

### Estimating Iron Atom Counts per Protein Species

Copy-number values for iron interacting proteins for each of 0h, 24h and 6d post-activation CD4+ and CD8+ T-cells were extracted from the Howden et al. dataset. If available, iron atom counts per protein were obtained using the Uniprot database cofactor information for each protein. Where iron atom counts were not available on Uniprot, estimates of iron usage per protein species was assumed to be 1 atom for heme and iron ion interacting proteins and 2 atoms for Fe-S cluster interacting proteins. The iron atom estimates per protein species were multiplied by the protein copy-number to produce estimates of iron atoms required by each protein population. The total number of iron atoms required per cell was calculated as the sum of iron atoms required by each protein species, while the “iron need” per cell was calculated as the difference in iron atoms per cell between 0h and 24h post-activation.

To stratify our iron count per protein species by iron interaction we utilised the iron interaction classifications provided by Andreini et al. (12). To stratify by cellular pathway, we utilised the gene sets for the GO terms: iron ion homeostasis (GO:0055072), DNA replication (GO:0006260), iron-sulfur cluster assembly (GO:0016226), oxidative phosphorylation (GO:0006119), aerobic respiration (GO:0009060), histone demethylation (GO:0016577) and DNA demethylation (GO:0080111). We combined the GO terms for oxidative phosphorylation and aerobic respiration due to discrepancies in both GO terms. Where overlaps in genes between GO term gene sets were identified, genes were allocated to the gene set deemed most appropriate: GLRX3, ISCU, ACO1 and NUBP1 were assigned to iron-sulfur cluster assembly and IREB2 was assigned to iron ion homeostasis.

### Modelling Iron Uptake Based on TSAT

TSAT values were derived from Tf and serum iron concentrations using the following equations from Yamanishi et al. (19):


$$\text{TSAT}\;\mathrm{(\%)}=\frac{[\text{Serum}\;\mathrm{Fe}]\left(\frac{\text{μmol}}{\text{L}}\right)}{\text{Total}\;\text{iron}\;\text{binding}\;\text{capacity}\;(\text{TIBC})\left(\frac{\text{μmol}}{\text{L}}\right)}\times 100$$

$$\text{TIBC}\left(\frac{\text{μmol}}{\text{L}}\right)=[\text{Tf},\;\text{g}/\text{L}]\times \frac{1\text{ mol}\;\text{Tf}}{795710\text{g}\;\text{Tf}}\times \frac{10^{6}\text{μmol}}{\text{mol}}\times 2\text{ Fe}\;\text{binding}\;\text{sites}$$

The following equations directly derived from Chasteen, et al. and Aisen, et al. (20, 21) were used to calculate the relative proportions of the 4 Tf forms given any TSAT value. **It should be noted that [Fe] is a RELATIVE unitless value and thus is only useful from within this set of equations.**

Relative association constants for Fe binding to the C and N termini of Tf


$$\begin{array}{l} {\text{k}}_{1\text{N}}^{'}=1\text{st}\;\text{atom}\;\text{N}\;\text{terminus}\;\text{binding}\\ {\text{k}}_{2\text{N}}^{'}=2\text{nd}\;\text{atom}\;\text{N}\;\text{terminus}\;\text{binding}\\ {\text{k}}_{2\text{C}}^{'}=1\text{st}\;\text{atom}\;\text{C}\;\text{terminus}\;\text{binding}\\ {\text{k}}_{2\text{C}}^{'}=2\text{nd}\;\text{atom}\;\text{C}\;\text{terminus}\;\text{binding} \end{array}$$

$${\text{X}}_{\text{A}}=\text{mole}\;\text{fraction}\;\text{of}\;\text{apoTf}=\frac{1}{1+({\text{k}}_{1\text{N}}^{'}+{\text{k}}_{1\text{C}}^{'})[\text{Fe}]+{\text{k}}_{1\text{N}}^{'}{\text{k}}_{2\text{C}}^{'}{\left[\text{Fe}\right]}^{2}}$$

$${\text{X}}_{\text{N}}=\text{mole}\;\text{fraction}\;\text{of}\;\text{N}\;\text{terminus}\;\text{monoTf}=\frac{1}{1+\frac{{\text{k}}_{1\text{C}}^{'}}{{\text{k}}_{1\text{N}}^{'}}+\frac{1}{{\text{k}}_{1\text{N}}^{'}[\text{Fe}]}+{\text{k}}_{2\text{C}}^{'}[\text{Fe}]}$$

$${\text{X}}_{\text{C}}=\text{mole}\;\text{fraction}\;\text{of}\;\text{C}\;\text{terminus}\;\text{monoTf}=\frac{1}{1+\frac{{\text{k}}_{1\text{N}}^{'}}{{\text{k}}_{1\text{C}}^{'}}+\frac{1}{{\text{k}}_{1\text{C}}^{'}[\text{Fe}]}+{\text{k}}_{2\text{N}}^{'}[\text{Fe}]}$$

$${\text{X}}_{\text{D}}=\text{mole}\;\text{fraction}\;\text{of}\;\text{diTf}=\frac{1}{1+\frac{{\text{k}}_{2\text{C}}^{'}+{\text{k}}_{2\text{N}}^{'}}{{\text{k}}_{2\text{C}}^{'}{\text{k}}_{2\text{N}}^{'}[\text{Fe}]}+\frac{1}{{\text{k}}_{1\text{C}}^{'}{\text{k}}_{1\text{N}}^{'}{\left[\text{Fe}\right]}^{2}}}$$

$$\text{TSAT}\;(\%)=50\;({\text{X}}_{\text{N}}+{\text{X}}_{\text{C}}+2{\text{X}}_{\text{D}})$$

Each molar fraction equation was substituted into the TSAT equation as follows:


$$\text{TSAT}\;(\%)=50[\frac{1}{1\;+\;\frac{{\text{k}}_{1\text{C}}^{'}}{{\text{k}}_{1\text{N}}^{'}}\;+\;\frac{1}{{\text{k}}_{1\text{N}}^{'}[\text{Fe}]}\;+\;{\text{k}}_{2\text{C}}^{'}[\text{Fe}]}+\frac{1}{1\;+\;\frac{{\text{k}}_{1\text{N}}^{'}}{{\text{k}}_{1\text{C}}^{'}}\;+\;\frac{1}{{\text{k}}_{1\text{C}}^{'}[\text{Fe}]}\;+\;{\text{k}}_{2\text{N}}^{'}[\text{Fe}]}\;+\frac{2}{1\;+\;\frac{{\text{k}}_{2\text{C}}^{'}+{\text{k}}_{2\text{N}}^{'}}{{\text{k}}_{2\text{C}}^{'}{\text{k}}_{2\text{N}}^{'}[\text{Fe}]}\;+\;\frac{1}{{\text{k}}_{1\text{C}}^{'}{\text{k}}_{1\text{N}}^{'}{\left[\text{Fe}\right]}^{2}}}]$$

The following literature values for relative association constants of iron for Tf were substituted into the equation which was rearranged and solved for the value [Fe] (20):


$${\text{k}}_{1\text{N}}^{'}=1.00\;{\text{k}}_{1\text{C}}^{'}=2.5\pm 0.30\;{\text{k}}_{2\text{N}}^{'}=0.66\pm 0.07\;{\text{k}}_{2\text{C}}^{'}=1.60\pm 0.30$$

Using the calculated [Fe] value → the values for X_A_, X_N_, X_C_, X_D_ could be determined, giving the relative molar frequencies of each Tf form. Using Tf concentration ranging from 1-4g/L, estimates of actual concentrations for each Tf form were calculated (22):


$$[\text{apoTf},\text{ mol}/\text{L}]=[\text{Tf},\text{ g}/\text{L}]\times {\text{X}}_{\text{A}}\times \frac{\text{mol}}{79570\text{g}\;\text{Tf}}$$

$$[\text{C}\;\text{terminus}\;\text{monoTf},\;\text{mol}/\text{L}]=[\text{Tf},\;\text{g}/\text{L}]\times {\text{X}}_{\text{C}}\times \frac{\text{mol}}{79570\text{g Tf}}$$

$$[\text{N}\;\text{terminus}\;\text{monoTf},\;\text{mol}/\text{L}]=[\text{Tf},\;\text{g}/\text{L}]\times {\text{X}}_{\text{N}}\times \frac{\text{mol}}{79570\text{g Tf}}$$

$$[\text{diTf},\;\text{mol}/\text{L}]=[\text{Tf},\;\text{g}/\text{L}]\times {\text{X}}_{\text{D}}\times \frac{\text{mol}}{79570\text{g Tf}}$$

To determine the relative probabilities of each Tf form binding to TFRC, literature values for association constants for Tf binding to the Tf receptor were used, substituting in the calculated concentrations for each Tf form (23).


$${\text{k}}_{\text{apo}}=\frac{4.6\times 10^{6}}{\text{M}}$$

$${\text{k}}_{\text{mono}\;\text{C}}=\frac{2.5\times 10^{7}}{\text{M}}$$

$${\text{k}}_{\text{mono}\;\text{N}}=\frac{2.8\times 10^{7}}{\text{M}}$$

$${\text{k}}_{\text{di}}=\frac{1.1\times 10^{8}}{\text{M}}$$

$${\text{k}}_{\text{apo}}=\frac{4.6\times 10^{6}}{\text{M}}\times [\text{apoTf},\text{ mol}/\text{L}]$$

$${\text{k}}_{\text{mono}\;\text{C}}=\frac{2.5\times 10^{7}}{\text{M}}\times [\text{C terminus monoTf},\text{ mol}/\text{L}]$$

$${\text{k}}_{\text{mono}\;\text{N}}=\frac{2.8\times 10^{7}}{\text{M}}\times [\text{N terminus monoTf},\text{ mol}/\text{L}]$$

$${\text{k}}_{\text{di}}=\frac{1.1\times 10^{8}}{\text{M}}\times [\text{diTf},\text{ mol}/\text{L}]$$

$$\text{P}({\text{k}}_{\text{apo}})=\frac{{\text{k}}_{\text{apo}}}{{\text{k}}_{\text{apo}}+{\text{k}}_{\text{mono}\;\text{C}}+{\text{k}}_{\text{mono}\;\text{N}}+{\text{k}}_{\text{di}}}$$

$$\text{P}({\text{k}}_{\text{mono}\;\text{C}})=\frac{{\text{k}}_{\text{mono}\;\text{C}}}{{\text{k}}_{\text{apo}}+{\text{k}}_{\text{mono}\;\text{C}}+{\text{k}}_{\text{mono}\;\text{N}}+{\text{k}}_{\text{di}}}$$

$$\text{P}({\text{k}}_{\text{mono}\;\text{N}})=\frac{{\text{k}}_{\text{mono}\;\text{N}}}{{\text{k}}_{\text{apo}}+{\text{k}}_{\text{mono}\;\text{C}}+{\text{k}}_{\text{mono}\;\text{N}}+{\text{k}}_{\text{di}}}$$

$$\text{P}({\text{k}}_{\text{di}})=\frac{{\text{k}}_{\text{di}}}{{\text{k}}_{\text{apo}}+{\text{k}}_{\text{mono}\;\text{C}}+{\text{k}}_{\text{mono}\;\text{N}}+{\text{k}}_{\text{di}}}$$

The weighted iron uptake and cycle time per TFRC protein was calculated as the probability of each Tf form binding to TFRC multiplied by the corresponding number of iron atoms or cycle time. The apoTf cycling time of 60 minutes was derived from Nuñez et al. (24). The diTf cycling time of 14.53 minutes was estimated as the average of cycling times described by 6 different methods in Nuñez et al. (24) and reviewed by Mayle et al. (25). monoTf cycling times were assumed to fall between diTf and apoTf cycling times at an intermediate 37.265 minutes as literature values could not be found.


$$\text{iron}\;\text{uptake}\;=\;\left(0\;\times \text{ P}({\text{k}}_{\text{apo}})\right)+\left(1\;\times \text{ P}({\text{k}}_{\text{mono}\;\text{C}})\right)+\left(1\;\times \text{ P}({\text{k}}_{\text{mono}\;\text{N}})\right)\;+\left(2\;\times \;P({\text{k}}_{\text{di}})\right)$$

$$\text{cycle}\;\text{time}\;=\;\left(60\text{ min }\times \text{ P}({\text{k}}_{\text{apo}})\right)+\left(37.265\text{ min }\times \;P({\text{k}}_{\text{mono}\;\text{C}})\right)\;+\left(37.265\text{ min }\times \text{ P}({\text{k}}_{\text{mono}\;\text{N}})\right)+\left(14.53\text{ min }\times \;\text{P}({\text{k}}_{\text{di}})\right)$$

The time required to uptake the calculated “iron need” was calculated using the Howden et al. average TFRC copy-number at 24h, the iron uptake and cycle time values:


$$\text{iron}\;\text{acquired}\;\text{in}\;1\text{h}\;=\;\text{TFRC}\;\text{copy}\;\text{number}\;\times \;\text{iron}\;\text{uptake}\;\times \;\left[\frac{60\text{ min}}{\text{cycle}\;\text{time}}\right]$$

$$\text{Time}\;\text{required}\;\text{to}\;\text{meet}\;\text{iron}\;\text{need}\;=\;\frac{\text{Iron need}}{\text{Iron}\;\text{acquired}\;\text{in}\;1\text{h}}$$

### Mice

Animal work was conducted under the authority of the UK Home Office project and personal licenses granted under the Animals (Scientific Procedures) Act 1986. Mice were housed in individually ventilated cages.

Inducible hepcidin knockout mice (iHampKO: *Rosa26-CreERT2 Hamp^flox/flox^*) were previously produced in Armitage et al. (26) and feature a fused Cre recombinase-estrogen receptor (CreERT2) protein under the control of a Gt(ROSA)25ser promoter and exons 2 and 3 of *Hamp1* located between LoxP sites. Mice carrying the floxed *Hamp1* loci but lacking the CreERT2 fusion peptide were used as controls (iHampCtrl: *Hamp^flox/flox^*). Administration of tamoxifen induces CreERT2 expression and subsequent *Hamp1* knockout in iHampKO mice but not iHampCtrl mice.

OT-I CD45.1 mice were obtained from Vincenzo Cerundolo, University of Oxford.
C57BL6/J mice were purchased from Envigo.

### Immunisation Model

OT-I CD8+ T-cells were adoptively transferred to iHampKO and iHampCtrl mice one day prior to immunisation. For the adoptive transfer, spleens of OT-1 CD45.1 mice were collected, homogenised through a 40 μm filter and treated with red cell lysis buffer. Flow cytometry was used to assess the frequency of OT-I CD8+ T-cells in the suspension which was then was diluted to a concentration of 50000 CD8+ T-cells/mL in PBS. 100 μL containing 5000 OT-I cells was injected intravenously per mouse. Mice were immunised subcutaneously with 100 μL of MVA-OVA at 1 x 10^8^ PFU/mL in PBS. At 2 days post-infection, 1 mg of tamoxifen (Sigma, T5648) per mouse was prepared in 90% corn oil and 10% ethanol and administered *via* intraperitoneal injection to induce iron loading in iHampKO mice. At day 7 post-immunisation, mice were euthanised using a rising concentration of CO_2_. Spleen and lymph nodes were collected for flow cytometry analysis. For serum analysis blood was collected by cardiac puncture in BD microtainer SST tubes (Beckton Dickinson). Liver was collected in RNAlater (Thermofisher Scientific, AM7020) for RNA analysis.

### qPCR

Sections of liver tissue (2-3 mm^3^) were homogenised in 700 μL RLT+ buffer using a TissueRuptor (Qiagen). RNA was extracted from 350 μL of the resulting lysate using the Qiagen RNeasy Plus Mini Kit (Qiagen, 74136). RNA concentration and quality was measured using a Nanodrop One machine (Thermofisher Scientific) and cDNA was generated *via* reverse transcription using the High capacity RNA-to-cDNA kit (Applied Biosystems/Thermofisher Scientific, 4388950). qPCR was conducted using the Taqman gene expression master mix (Applied Biosystems/Thermofisher Scientific, 4369016) and the TaqMan Gene Expression Assays for *Hamp1* (Applied Biosystems/Thermofisher Scientific, Mm04231240_s1) and the housekeeping gene *Hprt1* (Applied Biosystems/Thermofisher Scientific, Mm01545399_m1).

### Serum Biochemistry

Blood was collected in BD microtainer SST tubes (Beckton Dickinson), allowed to clot and then centrifuged at 8000 g for 5 minutes. Serum was stored at -80°C. Serum measurements were conducted with the MULTIGENT iron kit and the John Radcliffe Hospital, Oxford, UK on an Abbott Architect c16000 automated analyser (Abbott Laboratories).

### Th17 Cell Culture

Iron free media was prepared using RPMI-1640 media, 5% pannexin NTS iron free serum substitute (Pan biotech, P04-95080), 1% penicillin/streptomycin and 1% glutamine. Media was supplemented with defined concentrations of human holoTf (R&D systems, 2914-HT-001G/Sigma-Aldrich, T0665) ranging from 0.001-0.625 mg/mL. Additional human apoTf (R&D systems, 3188-AT-001G/Sigma-Aldrich, T1147) was added to maintain a constant total Tf concentration of 1.2 mg/mL.

Murine spleen and lymph nodes were sterilely dissected and homogenised through 40 μm filters using EasySep buffer (Stem cell technologies, 20144). Naïve CD4+ T-cells were isolated using the EasySep Mouse Naïve CD4+ T-cell isolation kit (Stem cell technologies, 19765) and the EasyPlate EasySep magnet (Stem cell technologies, 18102) with the manufacturers protocols. Cells were stained with cell trace violet (Invitrogen, C34557) prior to culture. Cells were plated at a density of 0.5 x 10^6^ cells/mL in iron free media with defined holoTf supplementation, 50 μM β-mercaptoethanol and 1 μg/mL α-CD28 (Biolegend, 102115) in 96 well plates pre-coated with 5 μg/mL α-CD3 (Biolegend, 100239) in PBS for 2-3 hours at 37°C. To induce Th17 polarisation, cultures were supplemented with 20 ng/mL IL-6 (Biolegend, 575702), 5 ng/mL hTGF-β1 (Biolegend, 781802), 5 μg/mL α-IFN-γ (Biolegend, 505802) and 5 μg/mL α-IL-4 (Biolegend, 504102). T-cells were cultured for 96-120h at 37°C, 5% CO_2_.

### Flow Cytometry

For analysis of leukocytes from the immunisation model, spleen or lymph nodes were macerated through 40 μm filters and treated with tris ammonium chloride red blood lysis buffer. Cells were transferred to 96 round bottom plates and washed with PBS.

For analysis of *in vitro* cultured Th17 cells, cell were transferred to round bottom plates. For intracellular cytokine staining, cells were stimulated with cell activation cocktail (1:500) (Biolegend, 423301), brefeldin A (5 μg/mL) (Biolegend, 420601) and monensin (2 μM) (Biolegend, 420701) for 5 hours prior to staining.

The cells were stained with 30 μL of surface stain prepared in PBS and incubated for 20 minutes on ice. Cells were fixed a using fixation buffer (Biolegend, 420801) for 20 minutes on ice or for nuclear staining, cell were fixed with FoxP3 transcription factor fixation buffer (eBioscience, 00-5523-00) for 1 hour on ice. Prior to intracellular staining, cells were permeabilised using perm/wash buffer (Biolegend, 421002) for 20 minutes on ice and then stained with 20-30 μL of intracellular stain in perm buffer. Samples were analysed on an Attune NxT flow cytometer (Thermofisher Scientific) or a BD Fortessa flow cytometer (BD biosciences).

Gating schemes can be found in **Figures S2F** and **S5E**.

### Data Analysis

Analysis was completed using Excel (Microsoft) and Prism software (GraphPad), Ensembl (17), Uniprot (16) and gProfiler (18) online programs as well as custom code written using the R programming language.

## Results

### Identifying Iron Interacting Proteins Differentially Expressed During T-Cell Activation and Differentiation

Our analysis utilised the Howden et al. dataset which consists of quantitative protein mass spectrometry (MS) data for murine CD4+ and CD8+ T-cells at 0 hours, 24 hours and 6 days post-activation. CD4+ and CD8+ T-cells cultured for 6 days were differentiated towards Th1 and cytotoxic T-lymphocytes (CTLs) respectively and protein expression data at all timepoints was reported by both copy-number and concentration (4). Notably, total cellular protein concentration increases by two to three-fold within 24h post-activation and continues to increase at 6 days (4). Howden et al. considered proteins with fold change values greater than 1.5 and p-values less than 0.05 as significantly differentially regulated; we also utilised these threshold values (4). In contrast to the Howden et al. dataset, the list of iron interacting proteins provided by Andreini et al. focused on human proteins (12). To cross reference the datasets, we compiled a corresponding list of mouse iron interacting proteins *via* searching for homologous proteins (see *Methods*).

Using a computational approach, the list of murine iron interacting proteins was cross compared against the T-cell proteomic profiles provided by Howden et al. Of the 9436 proteins detected in the Howden et al. dataset, 204 were identified as iron interacting proteins (**Supplementary File 2**). This corresponds to a frequency of iron interacting proteins of 2.16% (**Table 1**) which is approximately the frequency expected by chance alone (12). This suggests that there is no apparent detection bias for or against iron interacting proteins in this dataset. However, when stratifying proteins by iron interaction type (Fe-S cluster, heme group or iron ion), heme proteins were underrepresented as assessed by a chi-squared test. While the composition of the original Andreini et al. iron interacting protein list was 48%, 35% and 18% respectively for heme, iron ion and Fe-S clusters (**Table 1**), the Howden data set only contained 28% heme interacting proteins (12).

**Table 1:** Frequency of iron interacting proteins in the Howden dataset.

			Iron interacting proteins	Heme interacting proteins	Iron ion interacting proteins	Fe-S cluster interacting proteins	p-value
Andreini et al. (12) (human)			~2%	192/398 (48.24%)	139/398 (34.92%)	70/398 (17.59%)	-
All proteins detected by Howden et al. (4)			204/9436 (2.16%)	57/204 (27.94%)	90/204 (44.12%)	60/204 (29.41%)	<0.0001 †
DIFFERENTIALLY EXPRESSED (VS NAÏVE)	CD4+ 24h	copy-number	161/6842 (2.35%)	43/161 (26.71%)	71/161(44.10%)	50/161 (31.06%)	0.8764 ‡
		concentration	154/6248 (2.46%)	39/154 (25.32%)	72/154 (46.75%)	45/154 (29.22%)	0.7241 ‡
	Th1 6 days	copy-number	175/8032 (2.18%)	46/175 (26.29%)	78/175 (44.57%)	51/175 (29.14%)	0.9122 ‡
		concentration	146/6503 (2.25%)	44/146 (30.14%)	61/146 (41.78%)	44/146 (30.14%)	0.7877 ‡
	CD8+ 24h	copy-number	174/7440 (2.34%)	44/174 (25.29%)	77/174 (44.25%)	56/174 (32.18%)	0.6327 ‡
		concentration	143/6305 (2.27%)	42/143 (29.37%)	60/143 (41.96%)	44/143 (30.77%)	0.8333 ‡
	CTL 6 days	copy-number	181/8169 (2.22%)	47/181 (25.97%)	79/181 (43.65%)	58/181 (32.04%)	0.6991 ‡
		concentration	151/6457 (2.34%)	44/151 (29.14%)	68/151 (45.03%)	42/151 (27.81%)	0.8776 ‡

Frequencies for iron interacting proteins are calculated as a fraction of total proteins detected, frequencies of individual iron interactions (heme, iron ion and Fe-S clusters) are calculated as a fraction of iron interacting proteins. Statistics are chi-square tests for goodness of fit for the distribution of iron ion, heme and Fe-S cluster iron interacting proteins. † The chi-square test was calculated relative to the Andreini dataset (12). ‡ Chi-square tests are calculated relative to the complete set of iron interacting proteins detected in the Howden dataset (4).

Iron interacting proteins were also identified amongst proteins considered differentially expressed by copy-number or concentration during T-cell activation (0h *vs* 24h) or differentiation (0h *vs* 6 days) for CD4+ and CD8+ T-cells (**Supplementary File 2**). In all cases, approximately 2% of differentially expressed proteins were identified as iron interacting. When further broken down into heme, iron ion or Fe-S cluster interacting proteins, the frequencies did not deviate significantly from the frequencies observed amongst all detected iron interacting proteins (**Table 1**).

**Figure 1:**
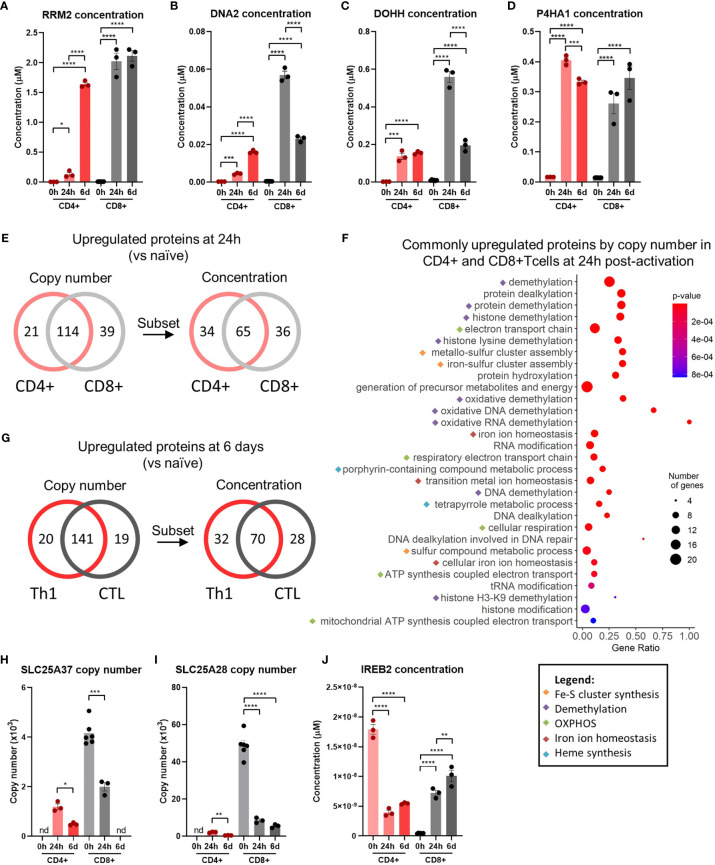
Iron interacting proteins are involved in diverse pathways during T-cell activation. An iron interacting protein list derived from Andreini et al. (12) was cross compared against the Howden dataset (4) consisting of protein-MS data for 0h, 24h and 6 day activated CD4+ and CD8+ T-cells. Protein concentrations for **(A)** RRM2, **(B)** DNA2, **(C)** DOHH and **(D)** P4HA1. **(E)** Number of differentially upregulated iron interacting proteins by copy-number and concentration between 0h and 24h for activated CD4+ and CD8+ T-cells and **(F)** GO term analysis of the 114 commonly upregulated iron interacting proteins by copy-number at 24h. Gene ratios indicate the percentage of gene hits within each GO term set. **(G)** Number of differentially increased iron interacting proteins by copy-number and concentration between 0h to 6 days for activated CD4+ and CD8+ T-cells. Protein copy number for **(H)** SLC25A37 and **(I)** SLC25A28 and protein concentration for **(J)** IREB2. Copy-number and concentration data is derived from the Howden et al. dataset (4). Data is mean ± SEM. Statistics for **(A–D, H–J)** are ordinary one-way ANOVAs with multiple comparisons using Tukey’s correction within CD4+ or CD8+ T-cells or in cases where absence of protein detection prevented use of one-way ANOVAs, unpaired t-tests with Welch’s correct were used. *p < 0.05; **p < 0.01; ***p < 0.001; ****p < 0.0001.

Ribonucleoside-diphosphate reductase subunit M2 (RRM2) was the most highly differentially upregulated iron interacting protein in CD4+ and CD8+ T-cells at both 24h and 6 days (**Figure 1A**). RRM2 is also amongst the top 10 highest expressed iron interacting proteins by absolute copy-number at 6 days post-activation in CD4+ and CD8+ T-cells (**Table 2**). RRM2 is a protein subunit of the ribonucleotide reductase (RNR) which catalyses the reduction step of deoxyribonucleotide synthesis and is essential for downstream DNA synthesis (27, 28). The di-iron centre of RRM2 is critical for RNR catalytic activity and iron chelation with desferrioxamine inhibits RNR activity in leukocytes (28, 29). Given the essentiality of nucleotide synthesis for DNA replication and proliferation, RRM2 appears to be a critical target for iron usage in T-cells.

**Table 2:** Top ten iron interacting proteins ranked by absolute copy-number.

RANK	CD4+	CD4+	Th1	CD8+	CD8+	CTL
	0h	24h	6 days	0h	24h	6 days
1	CYCS	ACO2	PPP1CA	HBB	PPP1CA	CYB5A
2	PPP1CA	GSTP1/2	CYB5A	GSTP1/2	GSTP1/2	PPP1CA
3	ACO2	PPP1CA	GSTP1/2	PPP1CA	CYCS	CYCS
4	CISD1	FTL1/2	CYCS	ACO2	ACO2	GSTP1/2
5	ABCE1	ADI1	BOLA2	FTL1/2	RRM2	ACO2
6	NDUFS1	CYB5A	ACO2	CYCS	FTL1/2	GLRX3
7	CISD2	GLRX3	CYB5B	CYB5A	BOLA2	BOLA2
8	SDHB	ABCE1	RRM2	CYC1	GLRX3	RRM2
9	GSTP1/2	NDUFS1	COPA	UQCRFS1	ABCE1	CYB5B
10	COPA	HBB	GLRX3	NDUFS1	PPAT	COX5A
Cumulative proportion of predicted total cellular iron content	67%	51%	41%	51%	37%	38%

Colours highlight proteins commonly found in the top 10 expressed iron interacting proteins between conditions.

DNA replication ATP-dependent helicase/nuclease (DNA2), Deoxyhypusine hydroxylase (DOHH) and Prolyl 4-Hydroxylase Subunit Alpha 1 (P4HA1) were also amongst the most highly upregulated iron interacting proteins and were the only other upregulated iron interacting proteins with fold changes greater than 15 in all subsets (**Figures 1B** **–D**). Similar to RRM2, DNA2 is involved in DNA replication and uses its Fe-S cluster to enable efficient DNA binding and mediation of helicase and nuclease activities (30). DOHH catalyses eIF5A hypusination from polyamines, a process critical for translational efficiency (31). eIF5A hypusination has been implicated in B cell function while polyamine availability and generation is critical for T-cell proliferation and viability (32, 33). P4HA1 is a proline hydroxylase most well-known for its role in collagen synthesis but is also known to hydroxylate other protein targets; the importance of P4H1 in T-cell function is unknown (34). The significant upregulation of these 4 diverse enzymes is testament to the widespread utilisation of iron in cellular function and indicates that iron deficiency may result in complex disruption of cellular activity.

Many of the iron interacting proteins identified by our analysis were differentially regulated in both CD4+ and CD8+ T-cells, highlighting a requirement for similar iron dependent processes. Of the iron interacting proteins that were significantly upregulated 24h post-activation, 114 proteins were found to be commonly upregulated in terms of copy-number in CD4+ and CD8+ T-cells (**Figure 1E**). 65 of these iron interacting proteins were also commonly upregulated in terms of concentration. Since the total protein content of T-cells increases upon activation and differentiation, for the concentration of these proteins to be significantly upregulated, the copy-number value must be experiencing a fold change increase greater than the fold change of total protein content. Thus, the proteins upregulated by concentration are a subset of proteins upregulated by copy-number and represent the most highly upregulated proteins. To understand if specific pathways may be particularly reliant on iron dependent proteins at 24h post T-cell activation, unranked pathway analysis using gProfiler (18) was completed on the sets of 114 iron interacting proteins upregulated by both CD4+ and CD8+ T-cells by copy-number at 24h post-activation (**Figure 1F**). The list of upregulated iron interacting proteins was enriched for GO terms relating to demethylation, Fe-S cluster synthesis, cellular respiration and unsurprisingly, iron homeostasis. The high enrichment of these pathways with iron interacting proteins, suggests that iron scarcity may disproportionately disrupt these processes.

At 6 days post activation, 141 proteins were commonly upregulated (from 0h) in both CD4+ and CD8+ T-cells by copy-number, of which 70 were also increased in concentration (**Figure 1G**). Pathway analysis for the 141 upregulated iron interacting proteins by copy-number produced a very similar list of iron interacting proteins to the enrichment at 24h (**Figure S1A**), indicating the continued necessity of iron dependent processes such as demethylation, OXPHOS and Fe-S clusters throughout T-cell activation and differentiation.

Downregulated iron interacting proteins were far less common (**Figure S1B, C**) but included iron interacting proteins such as albumin (ALB), hemopexin (HPX) and lysine demethylase 7a (KDM7A). Due to the low number of downregulated iron interacting proteins, pathway analysis on these proteins was not performed. However, we were also interested in identifying iron interacting proteins that displayed extreme differences in regulation upon activation between CD4+ and CD8+ T-cells. To do so, we filtered for proteins that showed significant differences in expression upon activation in CD4+ and CD8+ T-cells but in opposite directions. Using this method we identified three iron homeostasis proteins of interest (**Figures 1H–J**). Prior to activation, CD4+ T-cells showed no expression of either SLC25A37 and SLC23A28 (mitoferrins 1 and 2 respectively) which govern mitochondrial iron import (35, 36). In contrast, naïve CD8+ T-cells displayed detectable expression of both importers. Upon activation, CD4+ T-cells marginally but significantly upregulated both SLC25A28 and SLC25A37, while CD8+ T-cells dramatically downregulated both mitochondrial iron import proteins.

Iron response element binding protein 2 (IREB2) also showed divergent regulation in CD4+ and CD8+ T-cells (**Figure 1J**). IREB2 and aconitase 1 (ACO1) are proteins that post-transcriptionally regulate iron homeostasis (37). During cellular iron deficiency, IREB2 and ACO1 induce iron acquisition and retention by stabilising mRNAs that encode iron uptake proteins such as TFRC while blocking translation of mRNAs that encode proteins involved in iron sequestration and egress (37). While CD8+ T-cells increase IREB2 expression upon activation, CD4+ T-cells show downregulation of IREB2. Taken alone, this data may suggest that CD4+ T-cells have a reduced ability to respond to environmental iron signals relative to CD8+ T-cells. However, it should be noted that the significant downregulation of IREB2 in CD4+ T-cells may be partially or completely compensated by ACO1, whose concentration remains relatively constant in CD4+ T-cells at all time points (**Figure S1E**).

### Iron Scarcity Impairs Differentiation and Epigenetic Remodelling in Th17 CD4+ T-Cells

**Figure 2:**
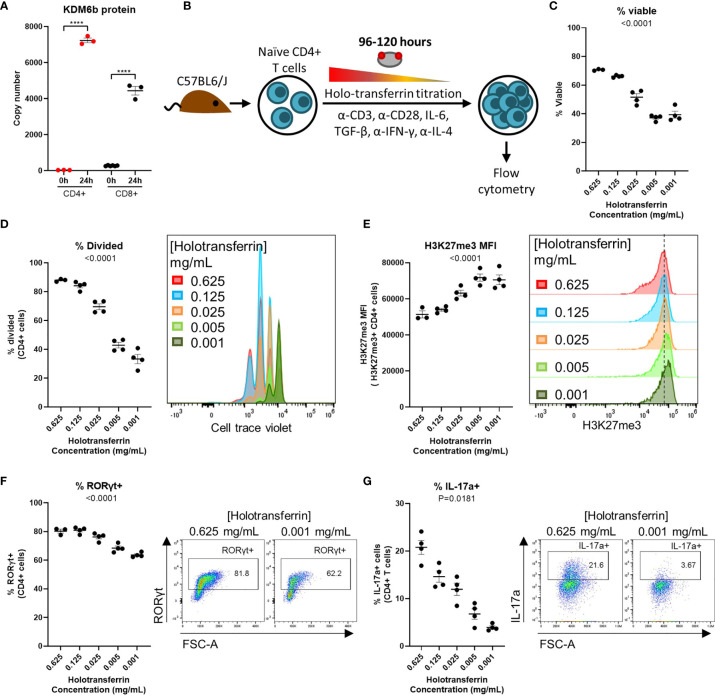
Iron deprived CD4+ T-cells in Th17-polarising conditions display impaired differentiation and epigenetic remodelling. **(A)** KDM6b protein copy number from the Howden et al. dataset (4). **(B)** Th17 iron deficiency model. Naive CD4+ T-cells were cultured in Th17 polarising conditions for 96-120h with holoTf titrated into iron free media at known concentrations. **(C)** Viability, **(D)** proliferation, **(E)** H3K27me3 MFI, **(F)** % RORγt+ cells, and **(G)** % IL-17a+ T-cells for cells cultured in Th17-polarising conditions. Graphs show mean ± SEM. An unpaired t-test was used for **(A)**, mixed effects analysis with repeated measures were used for **(C–F)** and a 1-way ANOVA was used for **(G)**. Data for **(C, D)** and **(E–G)** are representative of 3 and 2 experiments respectively. ****p < 0.0001.

Our computational analysis revealed that demethylation is a process highly enriched for iron interacting proteins during T-cell activation. The dependency of jumonji C domain lysine demethylases (KDMs) and ten-eleven translocation enzymes (TETs) on iron ion co-factors, suggests that iron-deficiency may unduly impair epigenetic remodelling (13, 38). Crucially, activating T-cells dramatically remodel their epigenetic landscapes to suppress expression of genes characteristic of naïve T-cells while permitting transcription of genes required for effector function (39, 40). Iron dependent KDMs showed dramatic changes upon activation in the Howden dataset (**Figure S2A**). KDM6b showed the greatest expression fold-change of all KDMs in CD4+ T-cells and the second greatest fold-change in CD8+ T-cells between 0h and 24h post-activation (**Figure 2A**). KDM6b is responsible for the removal of the repressive histone mark H3K27me3, a process critical for effector function acquisition and CD4+ T cell differentiation (39, 40). Notably, pharmacological inhibition of KDM6b was previously shown to attenuate Th17 CD4+ T-cell responses (39).

Given the importance of KDM6b for Th17 differentiation and the necessity of iron for KDM6b enzymatic activity, we hypothesised that iron starvation may alter T-cell epigenetic remodelling and in consequence Th17 differentiation. To assess the impact of iron deficiency on Th17 CD4+ T-cells we used an *in vitro* iron deficiency model of Th17 polarisation (**Figure 2B**) in which iron is titrated into iron depleted media in the form of iron-saturated transferrin (Tf), known as holotransferrin (holoTf). As iron availability decreased, CD4+ T-cells in Th17 polarising conditions showed significantly reduced viability and proliferation (**Figures 2C, D**). During iron deficiency, Th17 cells demonstrated elevated H3K27me3 expression (**Figure 2E**) indicating alterations in global chromatin remodelling. Elevated H3K27me3 expression in iron starved Th17 cells could be due to reduced passive loss of methylation by division dilution. However, we found that H3K27me3 levels were also increased in iron deprived T-cells that had not undergone division (**Figures S2B, C**), supporting our hypothesis that high H3K27me3 levels may be attributed to impaired active demethylation by KDM6 enzymes during iron deficiency. A concurrent decrease in the percentage of cells expressing the Th17 lineage defining transcription factor, RORγt, and cytokine, IL-17a, was also observed during iron starvation (**Figures 2F, G**). Cells that manage to divide at least once have some degree of differentiation advantage, as divided cells showed no difference in RORγt expression relative to iron replete controls (**Figures S2D, E**). This data shows that iron availability influences epigenetic regulation and differentiation of Th17 cells.

### Estimating T-Cell Iron Content Using Iron Interacting Protein Data

To better comprehend how alterations in iron availability may impact T-cell function, it is useful to assess the cellular iron requirements during activation and differentiation. Using protein copy-number data from the Howden dataset we estimated the average iron requirement of CD4+ and CD8+ T-cells at 0h, 24h and 6d post-activation given the assumption that all iron interacting protein iron binding sites are actively occupied. Where possible, known values of iron atoms per protein species were used based on searching the Uniprot database for iron cofactors (45% of iron interacting proteins in the Howden dataset, **Figure S3A**). In cases where exact values were not readily available, deliberate underestimations of 1 iron atom for heme or iron ion interactions or 2 iron atoms for Fe-S cluster interactions were used, to bias our estimates conservatively.

**Figure 3:**
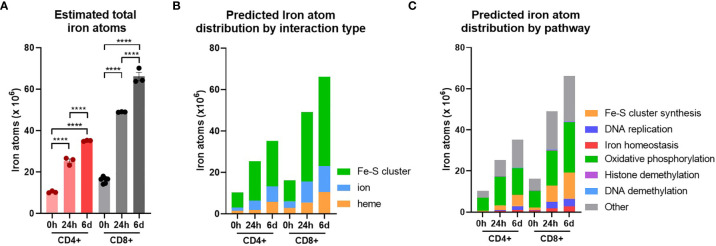
T-cells are predicted to increase total iron content upon activation. Cellular iron atom content was estimated by assuming complete saturation of all iron binding sites by iron interacting proteins. Where possible known values of iron atom:protein stoichiometry were used. If unknown, values were assumed to be 2 for Fe-S clusters, 1 for iron ions and 1 for heme groups. **(A)** Estimates for iron atom counts per cell. Estimates for iron atom distribution by **(B)** interaction type (Fe-S cluster, iron ion, heme group) and **(C)** pathway. Iron interaction information was derived from Andreini et al. (12). Pathways were defined using GO terms (see *Methods*). Data is mean ± SEM. Statistics for **(A)** are ordinary one-way ANOVAs with multiple comparisons using Tukey’s correction within CD4+ or CD8+ T-cells. ****p < 0.0001.

Naïve CD4+ and CD8+ T-cells had average iron atom estimates of ~10x10^6^ and 16x10^6^ respectively (**Figure 3A**). Estimates for iron requirements increase by ~2 fold for CD4+ T-cells and ~3 fold for CD8+ T-cells within the first 24h of activation and continue to increase at 6d post-activation. Howden et al. report a very large increase in the expression of the iron import protein, TFRC, throughout T-cell differentiation (3, 4). TFRC likely mediates the uptake of iron required to supply newly synthesised iron interacting proteins with their necessary iron cofactors.

To identify protein species which may collectively bind high amounts of iron due to a combination of high protein expression and/or high iron interacting protein:iron atom stoichiometry and thus act as “iron sinks”, we ranked iron interacting proteins by the predicted iron atoms bound by each protein species (**Table 3**). Amongst the protein species predicted to bind the most iron are ACO2, NDUFS1, SDHB and PPP1CA which were expressed at high levels in both CD4+ and CD8+ T-cells at all time points (**Table 3**). Strikingly, the top 10 proteins in each cell type and differentiation state are predicted to contain 48%-73% of all cellular iron.

**Table 3:** Top 10 iron interacting proteins ranked by predicted iron atoms per protein species.

RANK	CD4+	CD4+	Th1	CD8+	CD8+	CTL
	0h	24h	6 days	0h	24h	6 days
1	ACO2	ACO2	ACO2	ACO2	ACO2	ACO2
2	NDUFS1	NDUFS1	SDHB	NDUFS1	NDUFS1	NDUFS1
3	SDHB	SDHB	PPP1CA	SDHB	SDHB	SDHB
4	CYCS	GSTP1	BOLA2	HBB-BS	PPP1CA	HBB-BS
5	PPP1CA	NDUFS8	NDUFS1	GSTP1	PPAT	GSTP1
6	CISD1	PPP1CA	CYB5A	PPP1CA	NUBP2	PPP1CA
7	NDUFV1	PPAT	GSTP1	NDUFS8	BOLA2	NDUFS8
8	NDUFS2	NDUFV1	NUBP2	NDUFV1	GSTP1	NDUFV1
9	ABCE1	NUBP2	CYCS	FTL1	CYCS	FTL1
10	CISD2	GLRX3	NUBP1	CYCS	GLRX3	CYCS
Cumulative proportion of predicted total cellular iron content	73%	64%	52%	61%	48%	48%

Colours indicate iron interacting proteins commonly predicted to be amongst the top 10 that sequester iron between conditions.

When iron atom requirements are subdivided by iron interaction, iron atoms utilised in all iron interaction types (Fe-S clusters, ions and heme groups), increase upon activation (**Figure 3B**). At all stages of activation, over 60% of iron atoms in CD4+ and CD8+ T-cells are predicted to be involved in Fe-S clusters (**Figure S3B**). This is largely due to the nature of mammalian Fe-S clusters to contain two to four iron atoms per cluster (4Fe-4S, 3Fe-4S, 2Fe-2S) meaning that Fe-S cluster interacting proteins tend to have greater iron atom stoichiometry relative to heme or iron ion interacting proteins. We also observed the enrichment of Fe-S cluster synthesis proteins detected *via* GO term enrichment at 24h and 6d of activation (**Figures 1F** and **S1A**). Upregulation of Fe-S cluster synthesis machinery is required to facilitate the predicted high Fe-S cluster demand (**Figure 3B**).

While the GO term analysis enabled us to identify the most commonly differentially regulated pathways between T-cell subsets, it did not provide information as to the pathways that are most iron demanding. Using the gene set enrichment data, we identified GO terms of 6 different major iron requiring pathways. Using the gene sets for each term, we stratified our estimated iron counts per protein species by pathway. This analysis predicts that the largest proportion of iron atoms per T-cell are being utilised within OXPHOS (**Figure 3C**). In naïve T-cells, approximately 60% of iron atoms are localised in OXPHOS proteins. Following activation, the proportion of iron atoms in OXPHOS proteins, while still being the largest iron utilising pathway of the 6 we analysed, drops to approximately 40%. However, it should be noted that the absolute number of iron atoms in the OXPHOS pathway does increase with activation (**Figures 3C** and **S3C**). Notably, OXPHOS contains a high number of iron interacting proteins with relatively high stoichiometry of iron atoms per protein. For instance, NDUFS1 of CI contains 3 Fe-S clusters alone, corresponding to a total of 10 iron atoms. The majority of the iron interactions in the electron transport chain are with Fe-S clusters which partially accounts for the predicted high number of Fe-S cluster interactions within the cell (**Figure 3B**). Given the high demand for Fe-S clusters, Fe-S cluster synthesis was unsurprisingly predicted to have the second highest proportion of iron atoms. Moreover, the proportion of iron atoms in this pathway increases upon activation, again indicating the importance of Fe-S cluster synthesis during T-cell activation.

DNA replication was also a major hub of iron utilisation in 24h and 6d stimulated T-cells (**Figure 3C**). This is in agreement with the observation that RRM2, DNA2 and other iron requiring DNA replication enzymes (POLE, POLA1, PRIM2) were amongst the most upregulated iron interacting proteins post-activation. In contrast, while methylation was a top hit in gene enrichment analysis, both histone and DNA methylation make minor contributions of iron atoms to total predicted cellular iron content (**Figure 3C**). This discrepancy may be partially explained by the nature of demethylase iron interactions. While many of the enzymes involved in OXPHOS and DNA synthesis interact with Fe-S clusters containing two or more iron atoms, the JmjC KDM demethylases interact with singular iron atoms. Nevertheless, the prominence of JmjC KDM enzymes in our pathway analysis of commonly upregulated proteins is consistent with our *in vitro* data indicating the importance of iron for T-cell methylation remodelling (**Figure 2E**).

### Modelling T-Cell Iron Uptake Dynamics

The iron import protein, TFRC, is critical for immunological function and is upregulated upon T-cell activation (3–5). TFRC iron uptake is facilitated *via* binding to the serum iron binding protein transferrin which induces receptor mediated endocytosis (41). Once internalised, acidification of the endosome promotes iron release from Tf allowing for cellular use (41). TFRC is subsequently recycled back to the cell surface to complete an endocytic cycle (41).

Tf has two asymmetric iron binding sites located at the C and N termini of the protein with different iron binding affinities (20). Therefore, Tf can exist in 4 different forms depending on iron occupancy; apotransferrin (apoTf), C or N terminus monoferric Tf (monoTf), and diferric Tf (diTf), which bind to 0, 1 and 2 iron atoms respectively (20). Notably, all 4 forms are capable of binding and inducing endocytosis of TFRC, albeit with different affinities and with different endocytic cycling periods (23, 24). Since apoTf is also capable of binding and inducing endocytosis of TFRC, high levels of apoTf can effectively inhibit iron uptake *via* accumulation of TFRC within endosomes (24). Using equations developed by Aisen *et al*, the relative proportions of each Tf form can be calculated given the overall Tf saturation (TSAT) level (20, 21). Using known association constant values for TFRC binding to each Tf form we calculated the relative probabilities of TFRC binding to each Tf form (22, 23). With the calculated relative probabilities for TFRC-Tf binding, we estimated the average iron uptake and cycling time per TFRC protein for any given TSAT value.

**Figure 4:**
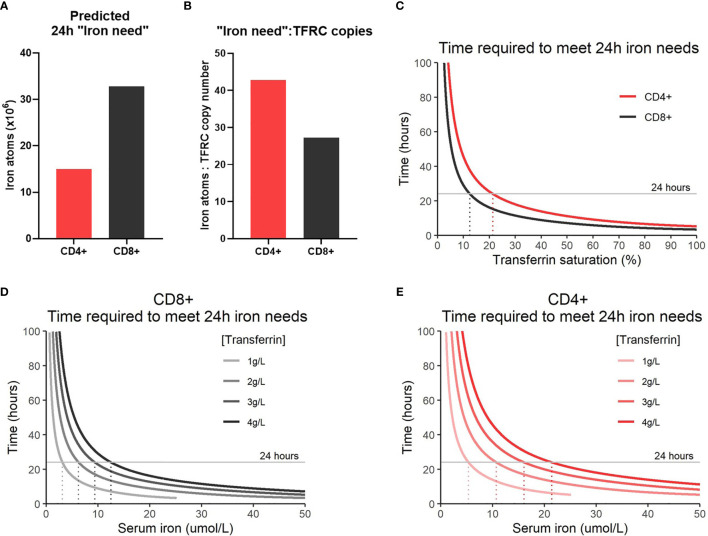
Modelling iron atom acquisition in the first 24h post-activation. **(A)** 24h “iron-need” and the **(B)** 24 “iron-need”:TFRC copy ratio. 24h “iron-need” is the difference in iron atom estimates between 0h and 24h. **(C)** Model of the time required for T-cells to acquire their 24h “iron need” *vs* TSAT. TSAT is a function of serum iron and Tf concentration. Models of the time required for **(D)** CD8+ and **(E)** CD4+ T-cells to acquire their 24h “iron need” at different serum iron concentrations.

Using this model, the time required to obtain the calculated 24h “iron need” was determined. “Iron need” was calculated as the difference in estimated iron atoms between 0h and 24h post activation (**Figure 4A**). It should be noted that our “iron need” predictions do not take into account intracellularly stored ferritin bound iron that may be released following activation. Ferritin levels are observed to increase in T-cells post-activation (**Figures S4A, B**) but how this influences access to cellular iron in T-cells is unknown. In our model, the rate at which T-cells are capable of taking up iron is a factor of TFRC expression. While CD4+ T-cells show reduced “iron need” relative to CD8+ T-cells, since CD4+ T-cells express lower levels of TFRC, this results in an increased “iron need”:TFRC ratio which is reflected in the slower iron uptake by CD4+ T-cells observed in our model (**Figures 4B, C**). The reduced rate of iron uptake by CD4+ T-cells in this model may indicate that CD4+ T-cells may be more sensitive to iron deprivation. Given that TFRC expression is at least partially driven by iron response proteins (IREB2 and ACO1) during T-cell activation (3), the lower ability to upregulate TFRC relative to predicted “iron need” in CD4+ T-cells may be due to the observed suppression of IREB2 in activated CD4+ T-cells relative to CD8+ T-cells.

In humans, TSAT values between 25-45% are considered normal, with TSAT values <16% defined as iron deficient by the World Health Organization (22, 42). In the context of inflammation, TSAT values <20% are often considered iron deficient (43). Our model predicts that T-cells will no longer be able to meet their iron requirements (over 24 hours) at TSAT values of ~10-20% (**Figure 4C**). This supports the idea that clinically defined iron deficiency is likely to impact on T-cell mediated immunity. Notably, our model demonstrates that the T-cells of individuals with TSAT values within the normal range should be able to easily meet iron requirements and it is unclear that there is any iron-acquisition benefit to T-cells with TSAT values greater than ~45%, for example as occurs in haemochromatosis and thalassaemia.

Given TSAT is derivable from Tf and serum iron concentrations, we were also able to model the rate of iron uptake based on these factors (**Figures 4D, E**). As Tf concentrations drop (as occurs physiologically during inflammation), our model predicts that the time required to meet cellular “iron need” also falls regardless of serum iron concentration. This is because suppression of Tf expression while iron concentrations remain constant effectively drives up the TSAT value and increases the probability of diTf : TFRC binding. Generally, our model indicates that suppression of serum iron levels may prevent T-cells from acquiring sufficient iron for activation needs, but that sensitivity of activated T-cells to low iron may be more pronounced in nutritional iron deficiency (in which transferrin levels are high-normal) compared to inflammatory hypoferremia (in which transferrin is low).

### Iron Overload Does Not Provide Significant Benefits to CD8+ T-Cells During Activation

Our mathematical model predicts that iron levels above the normal physiological range are unlikely to provide significant quantitative benefit to activating T-cells. To evaluate the impact of excess iron on T-cell immune responses and function, we utilised an inducible hepcidin knockout mouse [iHampKO: *Rosa26-CreERT2 Hamp^flox/flox^*, described previously (26)], which displays rapid serum iron loading following tamoxifen treatment. Hepcidin is a liver produced hormone that regulates systemic blood iron by inhibiting macrophage mediated iron recycling and dietary iron absorption (44); consequently, when its deletion is induced, iron is released to serum from macrophages and dietary iron absorption is enhanced leading to iron loading.

**Figure 5:**
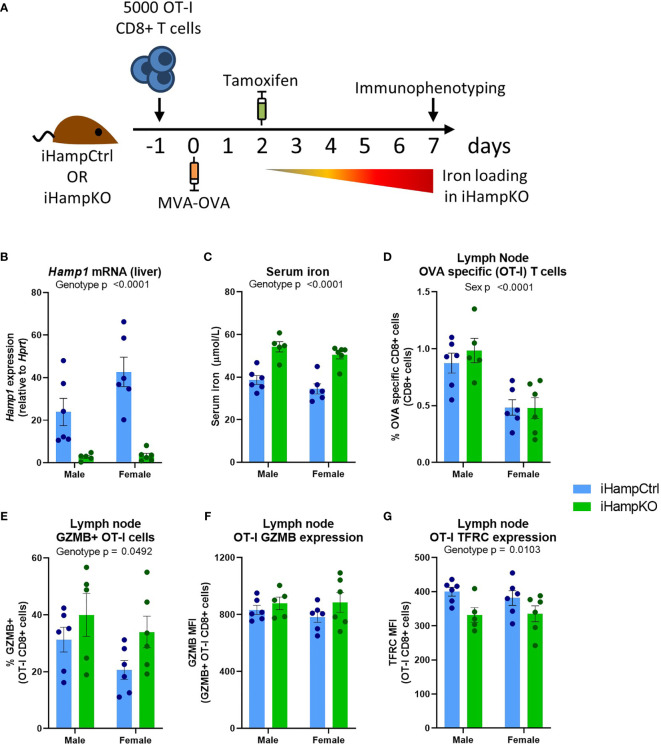
Induction of elevated serum iron does not significantly benefit activating T-cells. **(A)** Experimental setup. On day -1, 5000 OT-I CD8+ T-cells were adoptively transferred to iHampCtrl and iHampKO mice followed by immunisation with MVA-OVA at day 0. Iron loading was induced in iHampKO mice with tamoxifen on day 2 post-immunisation. **(B)** Liver *Hamp1* mRNA expression and **(C)** serum iron. Flow cytometry of lymph nodes was conducted at day 7 post-immunisation. Lymph node **(D)** OT-I frequency, **(E)** frequency of GZMB+ OT-Is, **(F)** GZMB MFI of GZMB+ OT-I cells and **(G)** OT-I TFRC median fluorescence index (MFI). Graphs shown mean ± SEM. Statistics for **(B–G)** are 2 way ANOVAs.

Ovalbumin (OVA) specific OT-I CD8+ T-cells were adoptively transferred to iHampKO or iHampCtrl (*Hamp^flox/flox^*) mice which were subsequently immunised with modified vaccinia virus Ankara-OVA (MVA-OVA) (**Figure 5A**) to induce a proliferative anti-OVA specific CD8+ T-cell response. Tamoxifen treatment was given at 2 days post-immunisation to induce *Hamp1* knockout resulting in elevated serum iron during the period of T-cell activation (**Figures 5B, C**). We then analysed the response of functionally wild type antigen specific T-cells in the context of systemic hepcidin deficiency and consequent iron loading. Consistent with our mathematical model, elevation of serum iron did not confer a proliferative benefit to T-cells. Comparable frequencies of OVA-specific T-cells were observed between iHampCtrl and iHampKO mice in both lymph nodes and spleen (**Figures 5D** and **S5A**). A higher frequency of OT-I cells expressing GZMB (a key mediator of cytolytic activity) was observed in lymph nodes (but not spleen) of iron loaded animals; the expression level [median fluorescent intensity (MFI)] of GZMB was not affected by iron loading (**Figures 5E, F** and **S5B, C**). TFRC expression is inversely proportional to cellular iron (45). Expression of TFRC by splenic and lymph node OT-I cells was slightly but significantly lower in iHampKO mice, consistent with activated OT-I cells acquiring more iron from a high serum iron environment (**Figures 5G** and **S5D**).

## Discussion

Using a combination of data mining from publicly available datasets and experimental methods, we investigated the dynamics of iron and iron interacting proteins during T-cell activation and differentiation. Our analysis indicates that T-cells rapidly and substantially increase iron demands post-activation for use in diverse cellular pathways including OXPHOS, demethylation and DNA synthesis. As evidence for the potential impact of iron deficiency on T-cell biochemistry, we show that iron depletion impairs removal of a key suppressive histone methylation mark and differentiation in an *in vitro* model of Th17 polarisation. In contrast, excess iron was shown to have no significant quantitative benefit for T-cell responses *in vivo* in comparison to control iron replete animals.

T-cell pathways enriched for iron interacting proteins such as DNA synthesis, OXPHOS, demethylation and Fe-S cluster biogenesis may be particularly susceptible to dysfunction during iron deficiency. Iron atoms in Fe-S clusters seem especially important as they make up the majority of predicted iron atoms per cell. Fe-S cluster biogenesis appeared as a top hit in pathway enrichment and showed an increase in the proportion of iron atoms in that pathway during T-cell activation. Further, Fe-S cluster synthesis feeds into many pathways including OXPHOS which was predicted to be the pathway with the highest concentration of iron atoms. *In vitro* iron deprived T-cells have been shown to suppress mitochondrial ATP generation, further reinforcing the necessity of iron for T-cell OXPHOS (3).

Using our computational analysis alone, it is difficult to evaluate the degree of impairment of iron deficiency on independent processes. For instance, histone and DNA demethylation were identified as critical iron dependent processes due to the high number of individual iron interacting proteins in these pathways. But, the absolute number of iron atoms attributed to DNA and histone demethylases is relatively low compared to OXPHOS and Fe-S cluster synthesis. However, using the computational analysis as a foundation, we predicted that KDM6b may be a major iron-demanding epigenetic regulator. Using experimental methods we show that iron deprivation impairs chromatin remodelling in Th17 cells with elevated expression of the KDM6b target, H3K27me3. While we cannot be certain that the increase in H3K27me3 is due to reduced demethylation by KDM6A/B rather than increased methylation, previous studies have indicated that inhibition of KDM6A/B does impair Th17 differentiation and CD8+ T-cell proliferation and memory formation (39, 46). In contrast, while DNA synthesis did not score amongst the highest enrichment terms in pathway analysis, two critical enzymes involved in DNA synthesis, RRM2 and DNA2, were amongst the most highly differentially regulated iron interacting proteins in activated T-cells, indicating that iron dependent DNA synthesis proteins are likely critical for T-cell activation. This is in agreement with previous data showing that *in vitro* iron deprivation impairs DNA synthesis and cell-cycle progression in CD8+ T-cells (3).

It is still unclear how iron depletion affects intracellular iron distribution. Data from the Howden dataset showed that Mitoferrins 1 and 2, which mediate mitochondrial iron transport, have different expression kinetics in CD4+ and CD8+ T-cells. Given that pathway analysis highlighted that iron dependent mitochondrial pathways such as OXPHOS, Fe-S cluster synthesis and heme synthesis are commonly upregulated in both CD4+ and CD8+ T-cells, it seems counterintuitive that mitochondrial iron transporters show different kinetics of expression in these two cell types. One may speculate that CD4+ and CD8+ T-cells may differentially store and re-distribute iron throughout the cell prior and during activation. The ability of different T-cell subsets to move iron into the mitochondria could profoundly affect the nature of iron interactions in the cell as the flux of iron into mitochondrial heme and Fe-S cluster biosynthetic pathways could present a major bottleneck for cellular iron usage.

Using protein copy-number values we estimated the total number of iron atoms per cell at various stages of activation and predicted that T-cell iron content increases 2-3 fold post-activation. The dramatic transition from quiescent naïve cells with relatively low estimated iron content to rapidly proliferating activated cells with elevated predicted iron content provides a rationale as to why cellular iron deficiency appears to more significantly impair activated versus naïve cells. Humans and mice carrying a TFRC mutation which impairs iron uptake have normal percentages of circulating naïve CD4+ and CD8+ T-cells, however, TFRC mutant T-cells fail to proliferate upon activation stimuli both *in vitro* and *in vivo* (3, 5). The quiescent state of naïve T-cells likely means that once adequate cellular iron is acquired, supply of iron to iron-dependent proteins can be maintained through internal turnover of iron binding proteins.

Measures of naïve T-cell iron have previously been evaluated using inductively coupled plasma mass spectrometry (ICP-MS) where iron measures for bulk T-cells were divided by the number of input cells (47). Our predicted iron content for naïve T-cells was approximately the same as the 75 percentile value for CD8+ T-cells and 1.5 fold higher than the 75 percentile value for CD4+ T-cells as measured by Konz et al. (47). While our values are on the high end of the values reported by Konz *et al*, they are of a similar magnitude giving us confidence in our methods of estimation.

Using the estimated iron counts per cell and existing kinetic data for Tf-TFRC endocytic cycling kinetics, we constructed a model to simulate T-cell iron uptake at various TSAT values. Our model predicts that at TSAT values between 10-20%, T-cells would no longer be able to acquire the iron they need to maintain occupancy of all iron binding sites. Low TSAT can occur either due to nutritional iron deficiency or due to inflammatory hypoferraemia caused by severe infections and/or chronic inflammation. For example, in SARS-CoV2 infection, several studies of hospitalised COVID-19 patients report average TSAT values of well below 15% with some patients with severe disease having TSAT values as low as 5% (48–51). Our model suggests that at such very low TSATs, T-cells are unlikely to be able to acquire sufficient iron for effective activation and differentiation. Suppression of Tf concentration from normal levels (2-3.5g/L) (22) has been observed during COVID-19 infection as well as other inflammatory states such as sepsis (48, 50–52). Suppression of Tf may be a compensatory mechanism to attempt to maintain TSAT homeostasis levels to preserve a constant rate of iron availability to the host immune system during hypoferremia.

Our model suggests that the economic theory of diminishing returns may be applicable to cellular iron nutrition: beyond a critical threshold, additional iron is unlikely to provide additional benefit. Once all iron binding proteins become saturated and are operating at peak rates, additional iron supply likely holds no advantage and may eventually become detrimental given the inherent toxicity of unchelated iron. The observation that T-cells activated in iron loaded conditions downregulate TFRC suggests that T-cells have an upper limit of desired cellular iron. In support of the non-linearity of our mathematical model, previous work from our group shows that serum iron restriction mediated *via* administration of a mini-hepcidin analogue significantly impairs both CD8+ and CD4+ T-cell proliferation and functionality in the context of diverse vaccination models and influenza infection (3). In contrast, we demonstrate that increasing serum iron beyond physiological levels *via* an inducible hepcidin knockout mouse model, does not enhance CD8+ T-cell proliferation or GZMB expression compared to iron replete control mice. Similarly, injection of wild-type mice on a standard diet with iron dextran, which increases serum iron, does not induce CD8+ T-cell proliferation beyond normal levels after immunisation (3). Recently, it has been reported that high iron concentrations may suppress CD4+ T-cell proliferation and Th1 differentiation (53), indicating that there may be complex context-dependent effects of excess iron on CD4+ T-cells. These data indicate that while iron supplementation may be able to boost immunity in individuals with existing iron deficiency, supplementing iron in iron replete individuals may provide little benefit.

### Limitations

One limitation to our bioinformatic approach relates to the input data. For instance, it was noted that the iron interacting protein SDHD was not detected in the Howden dataset. Given that SDHD is an essential component of complex II (CII) of the electron transport chain and all other CII proteins (SDHA, SDHB, SDHC) were detected, the absence of SDHD points to incomplete protein detection in the Howden dataset. Unfortunately, in most cases it is difficult to know whether lack of detection is due to biological or technical reasons limiting our capacity to account for undetected proteins when calculating T-cell iron content. Whether differences in the proportions of proteins involved in different types of iron interactions (heme, Fe-S cluster or iron ions) are inherent to the T-cell proteomic profiles or are due to a detection bias in the protein-MS method also remains to be determined.

When estimating T-cell iron content, we used known iron atom:protein stoichiometry values where possible. However, in cases where values were not readily available, we assumed low values of 1, 1, and 2 iron atoms for each of iron ion, heme and Fe-S cluster interactions respectively to minimise the probability of overestimating iron content. While known or cautious estimates for iron content can be made for most proteins, approximations for iron bound in ferritin complexes is difficult to assess given that ferritin cages can contain anywhere from 0 to ~4300 iron atoms (54). Ferritin levels do increase upon T-cell activation (**Figures S5A, B**), however, whether the iron content of ferritin cages changes with activation is also unknown. In our analysis, ferritin light and heavy chains (FTL1/2 and FTH1) were treated similarly to all other iron ion binding proteins and were assumed to bind one iron atom per protein. Thus, each ferritin complex is assumed to contain 24 iron atoms. Again, this is likely to result in a conservatively low approximation of T-cell iron content. It should be noted that the inability to properly assess ferritin iron content also impacts the predicted T-cell “iron need” as ferritin is likely to supply at least a fraction of the cellular iron requirements during activation. If naïve T-cells contain variable amounts of ferritin iron as a function of underlying iron status of the individual, this could influence the subsequent sensitivity of T-cells to extracellular iron sources following activation. Nevertheless, other evidence strongly supports a likely general dominant dependence of T-cell responses on extracellular iron. TFRC expression is upregulated over 200-fold following T-cell activation, and decreasing extracellular iron availability profoundly suppresses T-cell responses to immunization even in iron-replete animals (3, 4). Furthermore, a mutation in TFRC that reduces efficiency of extracellular iron uptake by ~50% causes severe immunodeficiency in children (5). While many of our assumptions aimed to bias our estimates conservatively, we also assume complete saturation of iron binding sites, likely overestimating iron binding per protein species. Perhaps as a result of these balancing assumptions we produced iron content estimates which are of a similar magnitude to experimentally observed values (47).

When building our model for T-cell iron uptake, we assumed that TFRC values increase instantaneously from 0h levels to 24h levels, whereas this process, although very rapid, will take longer. Neither does the model account for the increased TFRC expression that would occur in response to iron deficiency at low TSAT values. While the absolute relationship between TSAT and time required to meet T-cell iron needs may be inaccurate due to the reported limitations, the shape of the relationship is likely correct as evidenced by experimental data indicating that while low serum iron severely impairs T-cell responses (3), elevated serum iron has negligible beneficial effects (**Figure 5**).

In summary, using computational and experimental methods we have demonstrated the diverse nature of iron-interacting proteins in T-cell biology. We show evidence for effects of iron deficiency on epigenetic remodelling and T-cell differentiation, and describe the distinct impacts of iron scarcity and overload on T-cell responses.

## Data Availability Statement

The original contributions presented in the study are included in the article/**Supplementary Material**. Further inquiries can be directed to the corresponding author.

## Ethics Statement

Animal procedures were performed under the authority of UK Home Office project and personal licenses in accordance with the Animals (Scientific Procedures) Act 1986, and were approved by the University of Oxford ethical review committee.

## Author Contributions

MT designed the computational analysis. MT and JF designed and executed the experiments and analysed the resulting data. MT wrote the manuscript. AA and HD provided scientific guidance and supervised the project. All authors contributed to the article and approved the submitted version.

## Funding

This work was supported by the UK Medical Research Council (MRC Human Immunology Unit core funding to HD, award no. MC_UU_12010/10) and with the support of the Clarendon Fund and the Corpus Christi College A. E. Haigh graduate scholarship to MT.

## Conflict of Interest

The authors declare that the research was conducted in the absence of any commercial or financial relationships that could be construed as a potential conflict of interest.

## Publisher’s Note

All claims expressed in this article are solely those of the authors and do not necessarily represent those of their affiliated organizations, or those of the publisher, the editors and the reviewers. Any product that may be evaluated in this article, or claim that may be made by its manufacturer, is not guaranteed or endorsed by the publisher.

## Abbreviations

apoTf, apotransferrin; diTf, diferric transferrin; Fe-S cluster, iron sulfur cluster; GO, gene ontology; GZMB, granzyme B; H3K27me3, Histone 3 lysine 27 trimethylation; holoTf, holotransferrin; IL-17a, Interleukin 17; KDM, lysine demethylase; MFI, mean fluorescence intensity; monoTf, monoferric transferrin; OVA, ovalbumin; OXPHOS, oxidative phosphorylation; RORγt, RAR related orphan receptor gamma; Tf, transferrin; TFRC, Transferrin receptor; Th17 cells, T helper 17 cells; TSAT, transferrin saturation.
