# Supplementary material for: Analysis of Iron and Iron-Interacting Protein Dynamics During T-Cell Activation
Source: Front Immunol. 2021 Aug 12;12:714613. doi: 10.3389/fimmu.2021.714613 (PMC8647206; doi:10.3389/fimmu.2021.714613)
Supplement: Supplementary file 1 [file DataSheet_1.pdf]

Supplementary Material

1 Supplementary Figures and Tables

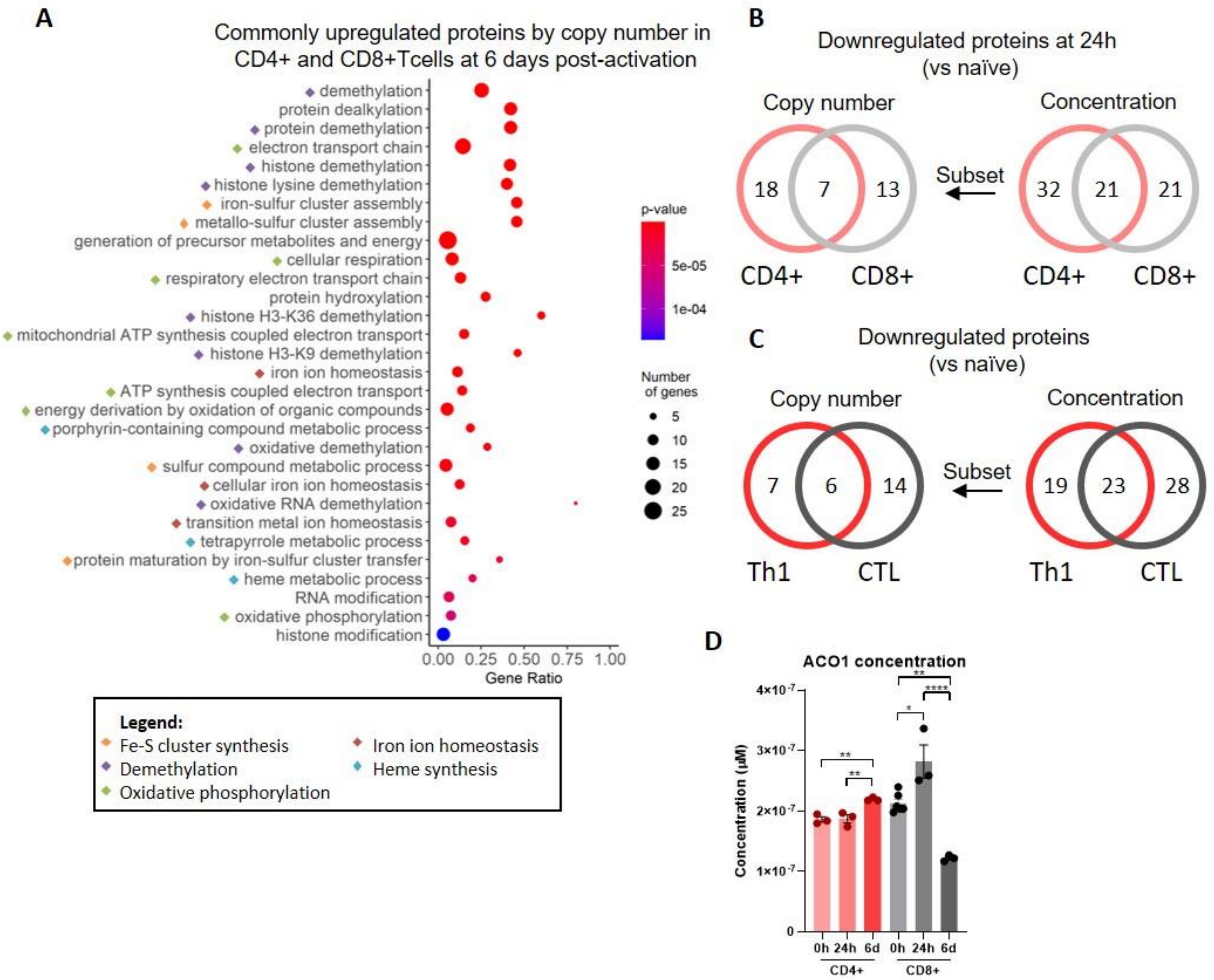

Figure S1. Identification of iron interacting proteins during T-cell activation. **(A)** Pathway analysis for iron interacting proteins commonly upregulated by copy-number on CD4+ and CD8+ T-cells at 6 days post-activation. Gene ratios are calculated as the frequency of gene hits within each GO pathway. Number of iron interacting proteins downregulated in CD4+ and CD8+ T-cell by copy-number and concentration at **(B)** 24h and **(C)** 6 days post-activation. **(D)** ACO1 protein concentration. Data shows mean ± SEM. Statistics for **(D)** are one-way ANOVAs with multiple comparisons using Tukey's correction within CD4+ or CD8+ T-cells. *$p < 0.05$; **$p < 0.01$; ****$p < 0.0001$.

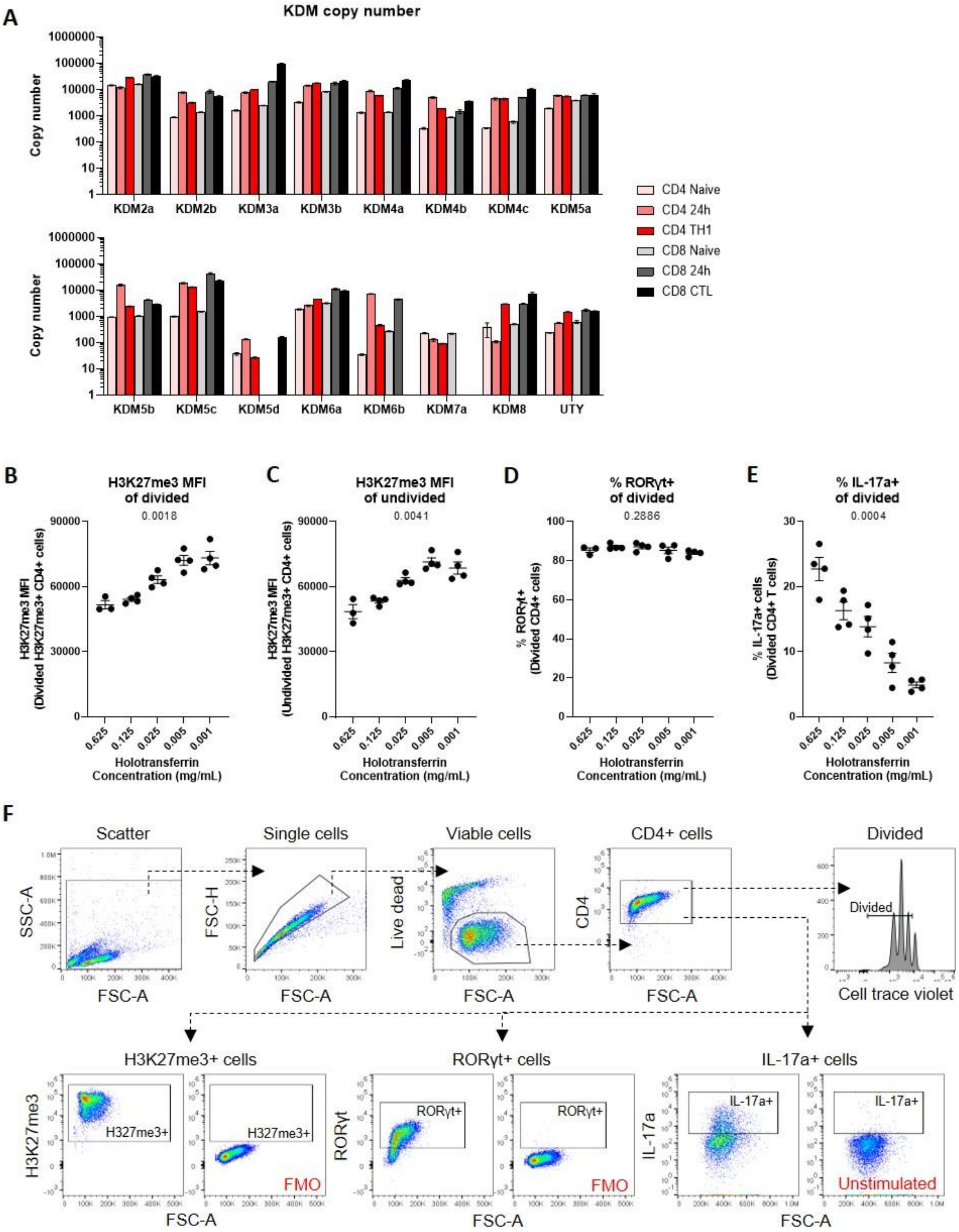

Figure S2. T-cell differentiation and epigenetic regulation is an iron dependent process. **(A)** KDM copy number in the Howden dataset (3). H3K27me3 MFI of **(B)** divided and **(C)** undivided CD4+ T-

cells cultured in Th17 polarising conditions. **(D)** % RORγt+, and **(E)** % IL-17a+ cells of divided Th17 polarised CD4+ T-cells. **(F)** Representative gating scheme for *in vitro* Th17 polarised cells. FMO = fluorescence minus one. Unstimulated cells were not treated with cell activation cocktail, brefeldin A or monensin. Graphs are mean ± SEM. Statistics for **(B-C)** are mixed effects analysis with repeated measures and statistics for **(D)** is a one-way ANOVA with repeated measures. Data for **(C-D)** are representative of 2 experiments.

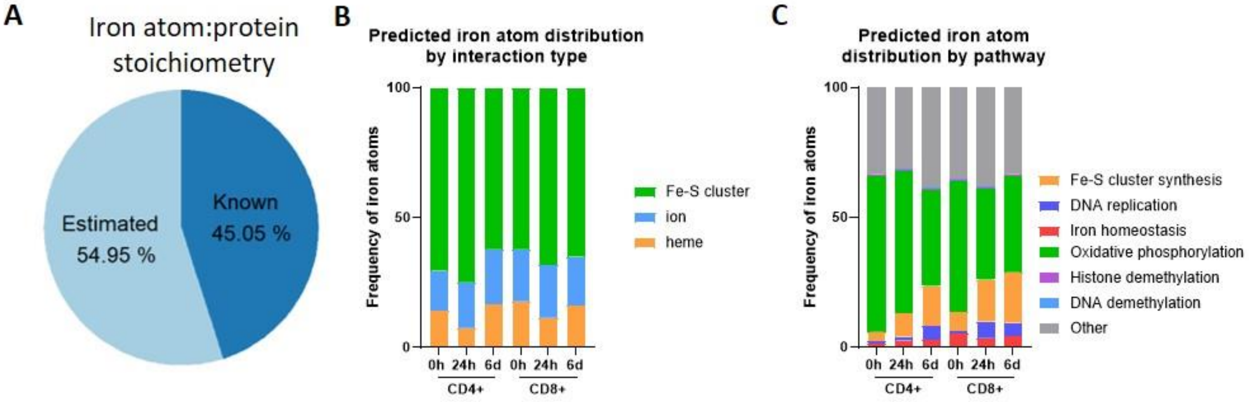

Figure S3. Estimating T-cell iron content and usage. **(A)** Frequency of proteins for which iron atom:protein stoichiometry was known vs predicted. Predicted frequency of iron atoms distributed by **(B)** interaction type and **(C)** pathway.

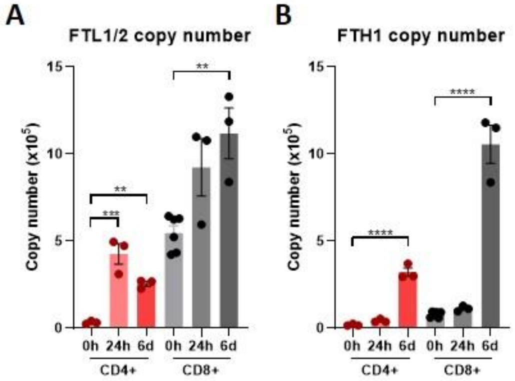

Figure S4. T-cells upregulate ferritin post-activation. **(A)** FTL1/2 and **(B)** FTH1 copy number in the Howden dataset (3). Data is mean ± SEM and statistics are one-way ANOVAs with multiple comparisons and Tukey's correction within CD4+ and CD8+ T -cells. **$p < 0.01$; ***$p < 0.001$; ****$p < 0.0001$.

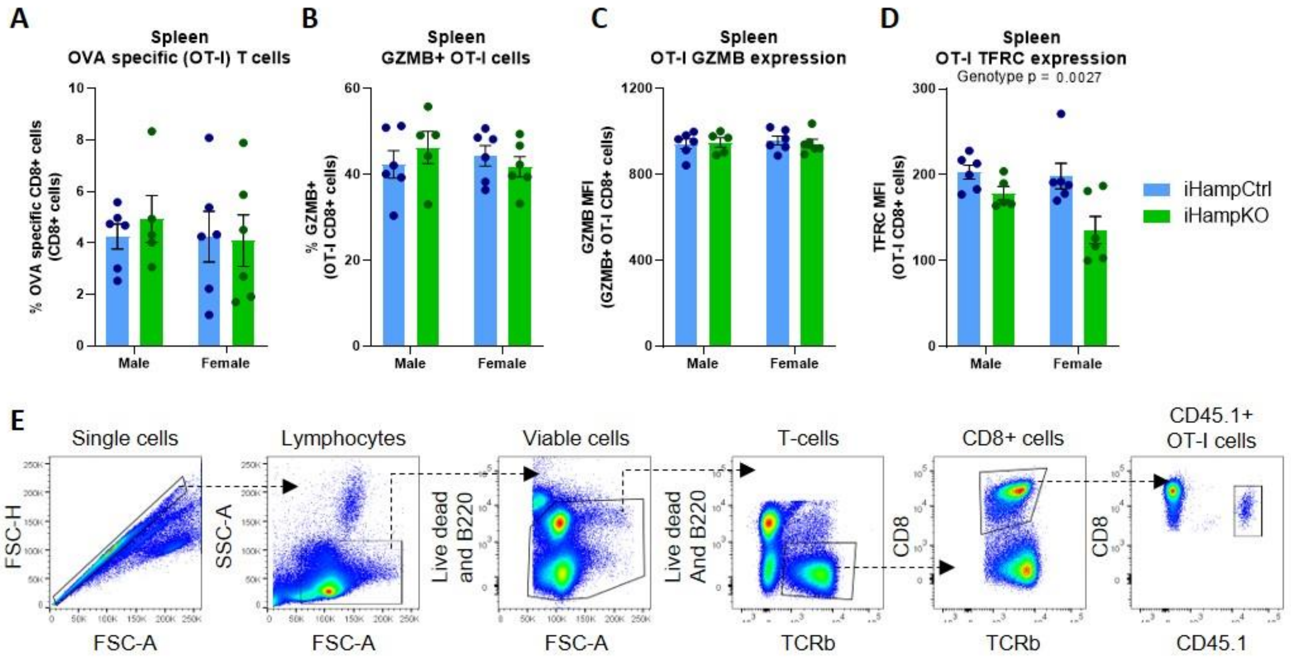

Figure S5. Hyperferremia does not improve splenic CD8+ T-cell responses. 5000 OT-I cells were adoptively transferred to iHampKO and iHampCtrl mice a day prior to immunisation with MVA-OVA. Tamoxifen was administered 2 days post-immunisation to induce serum iron loading and spleen cells were analysed at 7 days post-immunisation. Spleen **(A)** OT-I frequency, **(B)** % GZMB+ OT-I cells, **(C)** GZMB MFI of GZMB+ OT-I cells and **(D)** OT-I TFRC MFI. **(E)** Representative gating scheme for OT-I adoptive transfer/MVA-OVA immunisation model for spleen and lymph nodes. Data shown is for spleen. Graphs show mean ± SEM. Statistics for **(A-D)** are 2 way ANOVAs.

Supplementary table 1 – List of iron interacting proteins

Supplementary table 2 – List of extracted iron interacting proteins for each of the comparisons
